# Liver Transplantation and Aortic Valve Replacement

**Published:** 2011-02-01

**Authors:** H. R. Davari, S. A. Malek-Hosseini, H. Salahi, A. Bahador, S. Nikeghbalian, M. H. Nemati, M. A. Sanjarian, M. B. Khosravi, S. Shahbazi, M. Saberfiruzi, S. M. Dehghani, K. Kazemi

**Affiliations:** *Shiraz Organ Transplantation Center, Nemazee Hospital, Shiraz University of Medical Sciences, Shiraz, Iran*

**Keywords:** Cirrhosis, Severe aortic insufficiency, Orthotopic liver transplantation, Artic valve replacement

## Abstract

Surgical procedures involving heart and liver are rare and have been limited to either combined heart and liver transplantation or coronary artery bypass graft surgery (CABG) or aortic valve surgery and orthotopic liver transplantation (OLT). Aortic valve replacement (AVR) and pulmonary valve vegetectomy for bacterial endocarditis after OLT have also been reported. There are only five cases with aortic stenosis and cirrhosis reported to have combined AVR and liver transplantation. In the presence of cirrhosis, AVR has a significant risk for mortality because of bleeding from coagulopathy, renal failure, infection, and poor post-operative wound healing. Herein, we report on a case and management analysis of combined sequential AVR, and OLT in a 40-year-old cirrhotic man with Child and MELD score of C and 29, respectively. Echocardiography detected severe aortic insufficiency (AI) with enlarged left ventricle. Due to severe AI, the cardiologist recommended AVR prior to transplantation. The patient underwent metallic AVR. 4 months later, he received OLT. Both operations were successful and uneventful. Prioritizing AVR before OLT was successful in this patient. However, each patient must be evaluated individually and multiple factors should be assessed in pre-operation evaluation.

## INTRODUCTION

Multiple organ transplantation including combined liver and heart have been reported previously [1-8], but the number of these cases in the literature is few. Several cases of valvular heart disease secondary to bacterial endocarditis have been reported, that either had valve repair or replacement after orthotopic liver transplantation (OLT) [9-12]. In addition, five cases of combined aortic valve replacement (AVR) and OLT have been presented in the literature [1, 13, 14]. Herein, we report on the first case of AVR due to AI, followed by OLT for cirrhosis secondary to chronic hepatitis B infection.

## CASE PRESENTATION

A 40-year-old man with cirrhosis was evaluated at our institution for OLT in May 2003. He had been diagnosed as a case of chronic hepatitis B infection since 1998 and had clinical manifestations of end-stage liver disease (ESLD) such as encephalopathy, spontaneous bacterial peritonitis (SBP), and ascites during the last two years. He received a trial of ?α_1_ interferon (during mid-1998) and was put on lamivudine, spironolactone, propranolol, colchicine, and furosemide up to OLT. Liver biopsy was performed twice in 1998 and 1999—both showing severe chronic hepatitis. There was no past history of blood transfusion, surgery, or rheumatic fever. He had had an episode of pulmonary hemorrhage and bloody pleural effusion in October 2001. A thorough clinical and paraclinical evaluation was done. The patient had a Child Turrcotte Pugh score of 11 (C) and a MELD score of 29.

Due to coagulopathy, re-biopsy was not included in pre-operation evaluations. Abdominal computed tomography (CT) showed a small liver with significant irregularity of borders and coarse parenchymal density typical of ESLD. Color Doppler and abdominal CT revealed the portal vein to be patent. Transthoracic echocardiogram showed severe AI (3+), left ventricular end-systolic diameter (LVESD) of 50 mm, end-diastolic diameter (EDD) of 77 mm, and left atrial dimension (LAD) of 38 mm.

Following a thorough evaluation by cardiac and liver transplantation teams, we finally decided to perform AVR, before liver transplantation. Although there was a possible increased risk of hepatic encephalopathy because of cardiopulmonary bypass (CPB), the cardiologist recommended replacing the aortic valve at this stage. Bearing in mind that an excessive load will be on the heart during and after liver transplantation, the benefits of such a procedure outweigh its risks.

After correction of coagulopathy by administering fresh frozen plasma (FFP) and platelet, AVR was performed through median sternotomy in August, 2003. Intra-operatively, the cardiac surgeon found moderate left ventricular function with severe AI due to a perforated left coronary cusp. As the patient was young and a long survival was expected, a St. Jude metallic prosthetic valve number 23 was used. Total cardio-pulmonary bypass and aortic clamp times were 50 and 44 minutes, respectively. Aprotonin was administered during the operation. The AVR was successful. After transferring the patient to ICU, he was extubated on the same day and was discharged five days later.

On the ninth post-operative day, he was admitted again due to massive bloody pericardial effusion, impending to tamponade. Subxiphoid drainage was performed and again, FFP and platelet were administered to correct the coagulopathy. Despite all these efforts, the patient experienced a third admission due to bleeding from the operation site which was managed conservatively. 

During the interval between AVR and liver transplantation, platelet counts were always between 29,000 and 30,000×10^3^/µL; prothrombin time (PT) was measured to be 23 seconds (INR of 3.3), so no anticoagulation therapy was started.

Whole organ liver transplantation was performed in December 2003 (four months after AVR). Three liters of ascitic fluid and a cirrhotic liver with a patent portal vein were found during operation. The anhepatic phase was 52 minutes; the estimated blood loss during operation was 18,000 mL. The patient received 22 pints of packed red cell, 11 pints of FFP and seven pints of platelet. Aprotenin was administered continuously. Explanted liver pathology had mixed macro- and micro-nodular cirrhosis with portal vein thrombosis.

The liver transplantation was also uneventful, so the patient was extubated on the first post-operation day (PaO_2 _of 108.2 mm Hg; PaCo_2_ 30.3 mm Hg; and pH of 7.40; breathing nasal oxygen). The patient remained in ICU for five days. Immunosuppressive protocol included methyl prednisolone (1 g/day, IV) for the first three days, which was changed to oral prednisolone; cyclosporine (150–200 mg/day PO, started on the second day); and mycophenolate mofetil (Cellcept) (1 g/day with gradual increment to 2 g/day).

Echocardiography on the first post-OLT day showed an ejection fraction (EF) of 64% with normal myocardial function. Liver enzymes decreased steadily on the post-operative course. ALT decreased from 336 to 19 IU/L, AST from 195 to 68 IU/L, and alkaline phosphatase from 97 to 63 IU/L in less than a week. The patient experienced an episode of wound infection on postoperative day 10, which was debrided in operation room.

Echocardiography on the 20^th^ post-OLT day revealed an AVESD of 42 mm, with trivial AI; LVESD of 40 mm; and LVEDD of 52 mm; septal motion was flat; septum was hypokinetic; EF was 45%; LA dimension 44 mm; PAT was 60 ms; mean PAP was 50 mm Hg; RA was not dilated; but he had minimal pericardial effusion, and trivial mitral and tricuspid regurgitation.

In last follow up on August, 2010, the patient was found healthy. He was on tacrolimus 1.5 mg bid, mycophenolate mofetil (Cellcept) 500 mg q8h, atorvastatin and lamivudine. The patient also received warfarin with an INR maintained between 1.5 and 2.5.

In his last follow-up, he had a BUN of 22 mg/dL; Cr of 1.4 mg/dL; ALT 51 IU/L; AST 35 IU/L; alkaline phosphatase 298 IU/L; platelet count 101,000/µL; WBC of 4500/µL, triglyceride 139 mg/dL, and cholesterol 105 mg/dL. Sonoghraphy showed mild fatty liver infiltration. Echocardiography revealed an EF of 55%; LVESD 41 mm; LVEDD 65 mm; and LAD of 41 mm; normal functioning prosthetic aortic valve, and normal PAP.

## DISCUSSION

Liver transplantation in a one with cardiac disease exposes the patient to the risk of myocardial ischemia, peri-operative bleeding, further hepatic damage, renal dysfunction, and neurologic complications [15,16]. The potential for brain edema [17,18] and other hemorrhagic, hemodynamic, and metabolic complications, and poor wound healing is high when cardiac surgery precedes OLT [16]. However, there are few reports of two-stage surgical procedures (performed in two different sessions) when cardiac surgery preceded OLT [16,19] or when a simultaneous heart and liver transplantation was performed [20,21]. Combining a major cardiac surgery with liver transplantation greatly increases the risk of severe fibrinolysis and hemorrhage and the potential for hypotension and death. There has been no description of valvular cardiac surgery for AI preceding OLT, as it was with our case.

The peri-operative evaluation and anesthetic management of patients affected by severe liver disease and AI are of utmost importance. Theoretically, the key to a successful outcome is the selection and timing of the operative procedure that has the lowest peri-operative morbidity and mortality. This requires a clear understanding of the pathophysiology of both diseases.

It is well-known that patients with ESLD develop a hyperdynamic circulation, thought to be caused by pulmonary and porto-systemic shunts and circulating vasoactive substances [16]. The high cardiac output (CO) and increased preload results from a vasodilation-induced decrease in systemic pressure. On the other hand, intravascular volume may be depleted because of continuous formation of ascites and the diuretic therapy used to treat it. Moreover, paradoxically many cirrhotic patients have impaired contractile function and cardiomyopathy, due to production of false neurotransmitters, with norepinephrine depletion and catecholamine hypersensitivity. Overall, a cirrhotic patient’s heart has a limited ability to respond to stress as a result of maximal vasodilation, low intravascular volume, and low contractility.

In AI, preload reserve maintains cardiac output. However, in the later stages of the disease progression, any change in afterload may lead to impairment of ventricular performance even without any change in contractility. In addition, it is necessary to avoid significant reduction in preload that may be caused by venodilators or massive bleeding which may be seen in liver transplantation. It has long been recognized that postponing surgery for AI until the patient becomes severely symptomatic is potentially dangerous, as many patients will have already developed substantial ventricular enlargement and cardiac dysfunction by that time. Echocardiographic studies are routinely used to determine the appropriate timing for surgical intervention, necessitating AVR if severe AI is detected. 

The mental status of such a patient should be evaluated on admission, because of being prone to hepatic encephalopathy. The hemodilution associated with the use of CPB is likely to further decrease intravascular tonicity and colloid oncotic pressure, increasing intracranial pressure and the subsequent risk of fatal brain herniation [22,23]. Furthermore, cerebral blood flow during CPB may not be sufficient to provide adequate cerebral perfusion pressure in a setting of increased intracranial pressure.

We decided to perform AVR before OLT in order to have a better hemodynamic situation during liver transplantation. It seems to be reasonable, as we did an uneventful cardiac surgery without significant blood loss during operation. However, we had 18,000 mL blood loss during liver transplantation. The hemodynamic changes during liver transplantation were well tolerated possibly due to previous correction of AI. 

So far, there is no data demonstrating the effects of specific or combined AVR/OLT problems on morbidity or mortality of cirrhotic patients undergoing AVR. Nobuhiko Hayashida, *et al*, and Yong An, *et al*, reported a mortality rate of 100% to do cardiac surgery including AVR in a cirrhotic patient with Child C [24, 25].

The only problem that we encountered after cardiac surgery in our case was a bloody pericardial effusion impending to tamponade. It was secondary to generalized coagulopathy and responded well to subxiphoid drainage and supportive care. Liver transplantation was successful and the only post-operative complication was wound infection which was debrided in operation room. In subsequent follow-ups, hemodynamic status, cardiac and liver functions were acceptable.

We found no evidence of aortic root disease or congenital bicuspid valve. The cause of AI might be a subclinical bacterial endocarditis that had happened some yeas ago—most probably about the time of the pulmonary hemorrhage and bloody pleural effusion that had occurred. He was asymptomatic for AI because of the natural course of AI and hemodynamic changes such as decreased systemic vascular resistance due to liver cirrhosis.

There is still some controversy about the type of valve to be used. We used St. Jude metallic prosthetic valve. We considered long survival and young age as main factors for this selection, although the chance of endocarditis after transplantation and bleeding is higher with this type of valve. Use of bioprosthetic valves may be safer as it does not require anticoagulant therapy, thereby making multiple liver biopsies less hazardous; however, the risk of early structural valve deterioration is high [26]. We recommend every case to be individualized and decided separately.
